# Are Cytochrome P450 *CYP2C8* and *CYP2C9* Polymorphisms Associated with Ibuprofen Response in Very Preterm Infants?

**DOI:** 10.1371/journal.pone.0012329

**Published:** 2010-08-23

**Authors:** Xavier Durrmeyer, Shushanik Hovhannisyan, Yves Médard, Evelyne Jacqz-Aigrain, Fabrice Decobert, Jérome Barre, Corinne Alberti, Yannick Aujard, Claude Danan, Olivier Baud

**Affiliations:** 1 Neonatal Intensive Care Unit, CHI Créteil, France; 2 Neonatal Intensive Care Unit, Robert Debré Children's Hospital, Paris, France; 3 Department of Pediatric Pharmacology and Pharmacogenetics, CIC INSERM 9202, Robert Debré Children's Hospital, Paris, France; 4 Clinical Researches Functional Unit, CHI Creteil, France; 5 Clinical Epidemiology Unit, CIC-EC, Robert Debré Children's Hospital, Paris, France; 6 Fondation PremUP, Paris, France; Universidad Peruana Cayetano Heredia, Peru

## Abstract

**Background:**

Patent ductus arteriosus (PDA) in extremely preterm infants remains a challenging condition with conflicting treatment strategies. Ibuprofen is currently used to treat PDA with ductal closure failure rate up to 40%. We test the hypothesis that cytochrome P450 *CYP2C8/2C9* polymorphisms may predict ibuprofen response.

**Methodology/Principal Findings:**

We studied extremely preterm neonates with haemodynamically significant PDA and treated with ibuprofen. One or two variant *CYP2C8* and/or *2C9* alleles were found in 17% of the population, most of them were from Caucasian ethnicity (67–74%). Response to ibuprofen and clinical course of infants carrying variants *CYP2C8* and *CYP2C9* were similar. Comparing infants with wild type or variant *CYP2C8* and *CYP2C9* genotypes, response rate to ibuprofen was significantly higher in wild type than in mutated carriers in univariate analysis (73% versus 52%, p = 0.04). Comparing responders (ductus closure; n = 75) and non-responders (surgical ligation; n = 36), the only two factors significantly associated with the response to ibuprofen using multivariate analysis were higher gestational age and non Caucasian ethnicity but not CYP2C polymorphism.

**Conclusions:**

CYP2C polymorphism was not associated with PDA response to ibuprofen and this factor appears not appropriate to optimize the ductal closure rate by modulating ibuprofen dosing strategy. This study points out the role for ethnicity in the interindividual variability of response to ibuprofen in extremely preterm infants.

## Introduction

Patent ductus arteriosus (PDA) in very preterm infants born before 28 weeks'gestation remains a challenging condition regarding the treatment regimens and subsequent clinical consequences. PDA results in increased pulmonary blood flow and redistribution of flow to other organs responsible for several neonatal co-morbidities (ie, brain lesions, chronic lung disease, necrotizing enterocolitis). The patency of ductus arteriosus is observed in 55–70% of neonates born before 28 weeks' gestation, requiring medical or surgical closure [1]. In France, the cyclooxygenase inhibitor ibuprofen is currently used to treat PDA. However, failure of ductal closure is reported in up to 40% of infants treated with ibuprofen and may be more likely observed in the very immature neonates, leading to surgical ligation [2]–[4]. Even if surgery has limited complications, several adverse effects including pneumothorax, hypotension, intra-operative bleeding, phrenic nerve palsy, poor neurological outcome and death have been reported [5], [6]. Thus, pharmacologic treatment remains the first-line treatment [7].

There is still ongoing debate regarding the optimal dosage, schedule for ibuprofen administration in very preterm infants to improve ductal closure rate. The possible relationship between pharmacokinetic parameters and response to ibuprofen has been extensively investigated with conflicting results [8]. Ibuprofen serum concentration was found higher in patients with closed ductus arteriosus compared to patients with PDA after treatment [8], [9]. A higher dose regimen might achieve a greater closure rate; however, its tolerability and safety should be carefully evaluated as numerous adverse effects, including impaired renal function have been observed with ibuprofen treatment in neonates [10].

Another strategy might be to optimize the ductal closure rate by individualizing therapy regimen according to pharmacogenetic characteristics. Indeed, ibuprofen is a racemate of R- and S- enantiomers with 60% of R-ibuprofen being converted into S-ibuprofen. The racemic mixture undergoes stereoselective cytochrome P450 (CYP) dependant metabolism as CYP2C9 metabolizes S-ibuprofen and CYP2C8 metabolizes R-ibuprofen [11]. Both metabolic pathways are polymorphic under genetic control with large inter and intra-ethnic variability [11], [12]. Among 34 *CYP2C9* alleles, 2 mutated alleles, namely CYP2C9*2 and 2C9*3, were reported to have a significant impact on the metabolism of many drugs, including non steroid anti-inflammatory drugs and primarily ibuprofen and among others, antiepileptic, oral hypoglycaemic, and anticoagulant drugs. Similarly, among 14 *CYP2C8* alleles, CYP2C8*2 and CYP2C8*3 were associated with reduced enzyme activity *in vitro* [13]. In Caucasians, 22% of *CYP2C8* genes and 31% of *CYP2C9* genes have mutations with a linkage between the *CYP2C8* and *CYP2C9* genetic polymorphisms [14].

Data evaluating the pharmacokinetic consequences and clinical impact of ibuprofen polymorphic metabolism are available in adults but data in paediatric patients and primarily in neonates are lacking [14], [15].

Therefore, we tested here if individual *CYP2C8*/*2C9* genotypes may predict ibuprofen response in extremely preterm infants.

## Methods

### Patients and ethics

Extremely preterm infants with a gestational age below 28 weeks and admitted in the neonatal intensive care unit (Créteil Intercommunal Neonatal Intensive Care Unit) between March 2003 and April 2008 were prospectively entered into a standardized database. Gestational age was based on the date of the last menstrual period and on ultrasonographic findings during the first trimester of pregnancy. This study investigated 111 preterm infants with haemodynamically significant PDA (see below) and treated with ibuprofen, according to ductal response to treatment. In this monocentric study, all patients were treated and evaluated similarly along the study period. The study was approved by the local ethics committee (CCPPRB Henri Mondor), and written informed consent was obtained from the parents.

### Prospective data collection

We created a standard data collection (approved by local ethics committee) with parental informations, circumstances and route of the delivery, antenatal treatments (corticosteroid, antibiotics), neonatal morbidities (neonatal infection, respiratory distress syndrome, bronchopulmonary dysplasia (defined as oxygen and/or assisted ventilation requirement at 28 days of life and 36 weeks of postconceptional age), necrotizing enterocolitis), brain lesions observed on ultrasound. Standard neonatal cranial ultrasounds were performed using a 7.5 Mhz transducer (Vivid i ultrasound, GE Healthcare, Eilat, Israel). They were usually performed using a standardized scanning procedure, twice within the first week of life, around 21 days and thereafter at 36 weeks of gestational age. The main recorded events associated with preterm delivery were histological chorioamnionitis, premature rupture of amniotic membranes more than 24 hours before delivery, intrauterine growth retardation (IUGR, below 10^th^ percentile according to Kramer [16]) and preeclampsia. All outcomes, including death, were recorded until infants were discharged from the hospital.

### Criteria for PDA, ibuprofen treatment and evaluation of treatment response

The medical treatment criteria for patients were evaluated by colour Doppler ultrasonography using a 10 MHz probe (Vivid i ultrasound, GE Healthcare, Eilat, Israel). Echocardiographic criteria for PDA included evidence of left-to-right blood flow through the open ductus on color Doppler scanning, ductus diameter ≥1.4 mm/kg, increased left atrial diameter compared to aortic root (left-atrium-to-aortic-root ratio ≥1.4), and left pulmonary artery end diastolic flow velocity >0.2 m/sec as described by El Hajjar et al [17]. Ibuprofen-trometamol for parenteral use (Pedea® Orphan Europe laboratory, Paris- La Défense, France) was administrated. Three daily doses of ibuprofen of 10, 5 and 5 mg/kg were given intravenously over 15 minutes on 3 consecutive days at 24 hours intervals. Ibuprofen administration started between 12 and 72 h of life in most cases (n = 100) and was delayed in 11 remaining neonates. Infants with only a partially closed PDA after the first course of ibuprofen treatment underwent a second course 48 hours after the end of the first course. Responders were defined as patients without any clinical evidence of PDA and without luminal flow on Doppler examination 24 hours after the end of treatment regimen. Non responders underwent surgical ligation.

### *CYP2C8* and *CYP2C9* genotyping procedures

Blood samples were drawn after Institutional Review Board approval and informed parental consent were obtained. Genomic deoxyribonucleic acid (DNA) was extracted and kept frozen at −20°C until analysis.

The main enzymes involved in ibuprofen metabolism are *CYP2C8* and *CYP2C9*. For *CYP2C8*, the most common variant CYP2C8*3 alleles (rs10509681 and rs11572080 were tested but CYP2C8*2 was not tested. For *CYP2C9*, CYP2C9*2 (rs1799853) and CYP2C9*3 (rs1057910) were analyzed. All of the single nucleotide polymorphisms (SNPs) were detected after real time polymerase chain reaction (PCR) by the use of 5′nuclease allelic discrimination assays (ABI PRISM 7900HT Sequence detection system, Applied Biosystems, Foster City, CA). They were analyzed by commercial assays (TaqMan DME assays, Applied Biosystems) and the PCR conditions were identical to those of the manufacturer's instructions.

### Statistical methods

Results are expressed as median (range) for quantitative variables and absolute numbers (percentages) for qualitative variables. Comparisons between CYP genotype and between responses to treatment were performed with non parametric tests: Chi-2 or Fischer exact test as appropriate for qualitative variables, Wilcoxon test for quantitative variables. Patients were divided into two groups according to *CYP2C8* and *2C9* genotypes: patients homozygous wild type for both genes (wild type patients) and patients homozygous or heterozygous mutated for one or both genes (mutated patients).

Logistic regression model was used to assess the relationship between response to treatment and wild type, neonate sex, postmenstrual age, ethnicity and multiple pregnancies. Model selection used a stepwise backward-forward procedure. Results were expressed in bivariate analyses and at the last step of the stepwise procedure as odds ratios (OR) and 95% confidence interval (95% CI). Goodness-of-fit for the logistic regression model was tested using the Hosmer-Lemeshow chi-square test and discrimination by the *c*-statistic.

All tests were bilateral and level of statistical significance was set at 0.05. Statistical analyses were carried out using SAS 9.1 (SAS Inc, Cary, NC).

## Results

### Genotyping of neonates with PDA for CYP2C8 and CYP2C9 polymorphisms

Genotype frequencies for *CYP2C8* and *CYP2C9* (n = 111) are presented table 1. The frequencies of CYP2C8*1/*1, *1/*3 and *3/*3 genotypes were 83%, 16% and 1% respectively. The frequencies of alleles CYP2C9*1/*1(77%), *1/*2(15%); *1/*3(5%), *2/*2(1%), *2/*3(1%) and *3/*3(1%) did not differ significantly from those observed in previous reports [14], [18]. Most infants carrying the CYP2C8*3 allele were also carriers of CYP2C9*2. The number of patients carrying various combinations of both alleles (17/27) is higher than expected from the allele's frequencies suggested that there is a linkage between *CYP2C8* and *CYP2C9* variant alleles. No association between CYP2C8*3 and the other *CYP2C9* alleles was observed. Because different polymorphisms could induce different changes in ibuprofen pharmacokinetics and/or pharmacodynamics, we first analyzed clinical characteristics of CYP2C8*3 carriers compared with CYP2C9*3 carriers. No difference between these two groups was observed in both perinatal characteristics and ibuprofen response. Therefore, all mutated subjects were analyzed in the same group.

**Table 1:** Polymorphisms frequencies (N, (%)) of CYP2C8 and CYP2C9 in population (n = 111).

	**CYP2C8**				
		*1/*1	*1/*3	*3/*3	All
**CYP2C9**	*1/*1	84 (76%)	2 (2%)		86 (77%)
	*1/*2	2 (2%)	15 (14%)		17 (15%)
	*1/*3	5 (5%)			5 (5%)
	*2/*2			1 (1%)	1 (1%)
	*2/*3		1 (1%)		1 (1%)
	*3/*3	1 (1%)			1 (1%)
All		92 (83%)	18 (16%)	1 (1%)	

### Perinatal characteristics of neonates according to CYP2C8 and CYP2C9 genotyping

Tables 2 and 3 show the perinatal characteristics of neonates with PDA according to individual *CYP2C8* and *CYP2C9* genotypes (wild type n = 84 or mutated n = 27). Among our patients, statistically significant associations were observed between variant alleles for *CYP2C8* and *CYP2C9* and Caucasian ethnicity of both parents. In contrast, gestational age, birth weight, circumstances of preterm delivery and IUGR were not found associated with CYP genotypes. In postnatal period, we found a significant association between variant alleles carriers and a longer total ventilation duration (p = 0.01). However, there was no difference in chronic lung disease incidence or postnatal steroids use in this variant subpopulation. Severe brain lesions (high grades intraventricular haemorrhage and cystic periventricular leukomalacia) and neonatal mortality were not found different between the two groups.

**Table 2:** Perinatal characteristics of the population according to CYP genotype.

	Wild Type n = 84	Mutated n = 27	P
Male	44 (52%)	16 (59%)	0.53
Mother ethnicity			0.006
Caucasian	31 (37%)	18 (67%)	
Other	53 (63%)	9 (33%)	
Father ethnicity			0.001
Caucasian	32 (39%)	20 (74%)	
Other	51 (61%)	7 (26%)	
Caesarian section	40 (48%)	10 (37%)	0.33
Multiple pregnancies	23 (27%)	8 (30%)	0.82
Median birth weight (g), (range)	817	800	0.39
	(490–1290)	(530–1410)	
Median gestational age (weeks), (range)	26.3	26.0	0.07
	(24.3–27.9)	(24.1–27.7)	
Vascular placental disease	20 (24%)	4 (15%)	0.32
Preterm labour	65 (77%)	24 (89%)	0.19
Histological chorioamnionitis	41/80 (51%)	13/26 (50%)	0.91
Rupture of membranes >12 h	15 (18%)	5 (19%)	1.00
Antenatal betamethasone	79 (94%)	25 (93%)	0.68
IUGR<10 perc.	19 (23%)	5 (19%)	0.65

**IUGR: intra uterine growth retardation.**

**Table 3:** Clinical characteristics of the population according to CYP genotype.

	Wild type n = 84	Mutated n = 27	P
Additional surfactant	23 (27%)	13 (48%)	0.04
Haemodynamic support	13 (15%)	1 (4%)	0.18
Neonatal sepsis	9 (11%)	0 (0%)	0.11
Nosocomial sepsis	20 (24%)	3 (11%)	0.16
Median duration of total Ventilation	33.5	41.5	0.01
(days) (range)	(1.4–71.0)	(21.1–73.0)	
Median duration of mechanical ventilation(days) (range)	12.1(0.2–52.2)	18.9(0.6–73.0)	0.04
Median duration of non invasive ventilation(days) (range)	20.50.2–42.8	23.80–35.3	0.06
Chronic lung disease at 36 weeks	12 (15%)	4 (15%)	1.00
Postnatal systemic steroids	45 (56%)	17 (63%)	0.50
Inhaled steroids	50 (62%)	19 (70%)	0.42
Necrotizing enterocolitis	2 (2%)	2 (7%)	0.26
IVH all grades	20 (24%)	5 (19%)	0.57
IVH grades III-IV	3 (3%)	2 (7%)	NC
Cystic periventricular leucomalacia	1 (1%)	0	NC
Death before discharge	3 (4%)	0 (0%)	NC
**Response to ibuprofen**	**61 (73%)**	**14 (52%)**	**0.04**

**IVH: intraventricular haemorrhage.**

Surprisingly, response rate to ibuprofen treatment was found significantly higher in wild type carriers that in mutated carriers of *CYP2C8* and *CYP2C9* (73% vs 52%, p = 0.04). This result was unexpected with regard to the potential pharmacokinetics consequences of *CYP2C8* and *CYP2C9* variant alleles on the clearance of ibuprofen. Therefore, subsequent analysis of the cohort compared responders to non responders' neonates to ibuprofen treatment to investigate potential confounding variables mitigating any effect of *CYP2C8* and *CYP2C9* variants.

### Perinatal characteristics of neonates according to the response to ibuprofen treatment

Tables 4 and 5 describe the perinatal characteristics of neonates with PDA according the response to ibuprofen treatment. Among the cohort of patients, statistically significant associations were observed between non responders and Caucasian ethnicity of the mother (p = 0.01), multiple pregnancies (p = 0.03), birth weight (p = 0.009) and a lower gestational age (p<0.0001). In contrast, circumstances of preterm delivery and IUGR were not found associated with clinical response to ibuprofen. As expected in postnatal period, non responders required mechanical ventilation 3-fold longer than responders and chronic lung disease incidence and postnatal steroids use were higher in non responders patients (p = 0.02 and p = 0.001, respectively). Severe brain lesions (high grades IVH and cystic periventric`ular leukomalacia) and neonatal mortality were found not different between the two groups. In bivariate analyses, responders patients were found more likely to carry wild type genotype for *CYP2C8* and *CYP2C9* compared to non responders (81% vs 64%, p = 0.05). Using multivariable analysis, higher gestational age at birth (OR 2.6 95%CI 1.6–4.1) and non Caucasian ethnicity (OR 3.9 95%IC 1.5–10.1) (but not CYP genotype) were found to be statistically associated with the response to ibuprofen in extremely preterm neonates with PDA (Table 6).

**Table 4:** Perinatal characteristics of the population according to the response to Iboprofen.

	Responders n = 75	Non responders n = 36	P
Male	44 (59%)	16 (44%)	0.16
Mother ethnicity			0.01
Caucasian	27 (36%)	22 (61%)	
Other	48 (64%)	14 (39%)	
Father ethnicity			0.07
Caucasian	31 (41%)	21 (60%)	
Other	44 (59%)	14 (40%)	
Caesarian section	35 (47%)	15 (42%)	0.62
Multiple pregnancies	16 (21%)	15 (42%)	0.03
Median birth weight (g)	880	768	0.01
(range)	(490–1410)	(490–1170)	
Median postmenstrual age, weeks	26.6	25.6	<0.0001
(range)	(24.4–27.9)	(24.1–27.9)	
Vascular placental disease	19 (25%)	5 (14%)	0.17
Preterm labour	57 (76%)	32 (89%)	0.11
Histological chorioamnionitis	34/70(49%)	20/36(56%)	0.50
Rupture of membranes >12 h	16 (21%)	4 (11%)	0.19
Antenatal betamethasone	69 (92%)	35 (97%)	0.42
IUGR<10 perc.	17 (23%)	7 (19%)	0.70

**Table 5:** Clinical characteristics of the population according to the response to Ibuprofen.

	Responders n = 75	Non responders n = 36	P
Additional Surfactant	20 (27%)	16 (44%)	0.06
Haemodynamic support	11 (15%)	3 (8%)	0.54
Neonatal sepsis	5 (7%)	4 (11%)	0.47
Nosocomial sepsis	14 (19%)	9 (25%)	0.44
Median duration of total ventilation (days) (range)	28.6(1.4–62.9)	46.3(10.4–73.0)	<0.0001
Median duration of mechanical ventilation (days) (range)	6.9(0.2–35.5)	22.2(3.4–73.0)	<0.0001
Median duration of non invasive ventilation (days) (range)	20.6(0.2–41.7)	23.2(0–42.8)	0.16
Chronic lung disease at 36 weeks	7 (9%)	9 (26%)	0.02
Postnatal systemic steroids <36weeks	36 (49%)	26 (76%)	0.007
Inhaled steroids	44 (59%)	25 (74%)	0.16
Necrotizing enterocolitis	0 (0%)	4 (11%)	0.01
IVH all grades	16 (21%)	9 (25%)	0.67
IVH grade III-IV	2 (2%)	3 (8%)	NC
Cystic periventricular leucomalacia	1 (1%)	0 (0%)	NC
Death before discharge	1 (1%)	2 (6%)	0.24
**Wild Type CYP2C8 and 2C9**	**61 (81%)**	**23 (64%)**	**0.04**

**Table 6:** Bivariate and Multvariable logistic regression modelling of factors related to treatment response (OR: Odd Ratio, CI95%: 95% Confidence Interval).

Variable	Bivariate analyses			Multivariable analyses		
	OR	95% CI	p	OR	95% CI	p
Postmenstrual age (weeks)	2.4	(1.5–3.7)	<10^−3^	2.6	(1.6–4.1)	<10^−3^
Ethnic group						
Caucasian	1					
Other	3.0	(1.3–6.8)	<0.01	3.9	(1.5–10.1)	<0.01
CYP genotype						
Mutated	1					
Wild type	2.5	(1.0–6.0)	0.05			
Multiple pregnancies	0.4	(0.2–0.9)	0.03			
Sex						
Male	1					
Female	0.6	(0.3–1.3)	0.16			

*c*-statistics = 0.78, Hosmer and Lemeshow Goodness-of-Fit Test p = 0.82.

## Discussion

We have hypothesized that, in addition to prematurity and environmental factors, efficacy of ibuprofen to treat PDA was related to pharmacogenetics factors affecting ibuprofen metabolism and pharmacokinetics. The goal of this study was to determine the contribution of these pharmacogenetic factors to ibuprofen response and the potential relevance of genotype-based dosing of ibuprofen likely to increase PDA closure rate and subsequent neonatal morbidity.

Relationship between area under the curve and ibuprofen efficacy for PDA closure was reported in several studies [10], [18]. Therefore, it might be expected that genetic polymorphisms reducing ibuprofen metabolism would increase drug effectiveness. Interindividual variability in drug metabolism may account for clinical differences in therapeutic efficacy or adverse effects associated to ibuprofen treatment.

The CYP2C9 enzyme plays a key role in the formation of oxidative metabolites of both R-(inactive −) and S-(active +) ibuprofen [11], [19]. *In vitro* studies indicate that stereoselectivity of ibuprofen hydroxylation by human CYPs exists, CYP2C8 being the main enzyme involved in the hydroxylation of R-(−)-ibuprofen and CYP2C9 being the main enzyme involved in the hydroxylation of S-(+)-ibuprofen [19]. In adults, low ibuprofen clearance occurring is strongly linked to *CYP2C8* and *CYP2C9* polymorphism [14], [20].

We tested the major *CYP2C8* and *CYP2C9* allelic variants known to reduce ibuprofen clearance in healthy adults [2], [14] but our findings do not support the hypothesis that clinical response to ibuprofen could be related to *CYP2C8* and *2C9* genotypes in extremely preterm infants.

Variant alleles frequencies observed in the present study were consistent with published data. CYP variants were described more frequently in Caucasian than in non Caucasian populations [21]. In Caucasians, reported CYP2C allele frequencies were 9.5 to 17% for CYP2C8*3 and 16 to 21% for CYP2C9*2 [18] as compared to 17% for both of these alleles in our study. In agreement with previous findings, a gene linkage between CYP2C8*3 and CYP2C9*2 alleles was observed.

The absence of association between CYP2C polymorphisms and response to ibuprofen in extremely preterm infants might be related to other several factors:

- Despite a monocenter study design, the studied population remains heterogeneous in terms of preterm birth circumstances and severity of respiratory disease, two factors involved in the severity and clinical consequences of PDA. Moreover, the sample size of the studied population remains quite low.
- Pharmacogenetic expression of polymorphic enzyme activities may be developmentally influenced [22]. CYP2C protein expression and/or catalytic activity are most probably very low during the immediate postnatal period in very low birth weight infants. Indeed, *CYP2C9*-specific content and catalytic activity were consistent with expression at 1 to 2% of mature values during the first trimester of pregnancy, with progressive increase during the second and third trimesters to reach approximately 30% of mature values [23]. However, Koukouritaki et al. and others demonstrated that CYP2C maturation depends only on postnatal age and 51% of newborns exhibiting values close to mature levels suggesting that the most immature neonates could express a substantial amount of CYP [24].
- CYP 2C8/9 has been identified as an endothelium-derived hyperpolarizing factor (EDHF) synthase who is a physiologically relevant generator of reactive oxygen species (ROS) in vascular endothelial cells and modulates both vascular tone and homeostasis [25]. A decreased expression of *CYP2C* could reduce ROS accumulation and subsequent PDA closure [26].
- Some other ibuprofen metabolites may influence the clinical response of this drug deserving further testing through metabolomics studies [27].
- Finally, *CYP2C8* was found involved in the metabolism of arachidonic acid to biologically active epoxyeicosetrenoic acids (EETs) in the kidney. Indeed, CYP2C8*3 variant was associated with a reduced turnover of arachidonic acid to11,12-EET and 14,15-EET [13]. These epoxides have significant physiologic role in water reabsorption and Na+ transport, inflammation, and vascular smooth muscle tone [28]–[30] all factors likely to modulate vascular tone and to alter ductus closure.

In addition to CYP2C polymorphism, other genetic factors and ethnicity could also mitigate the impact of CYP2C genotype on response to ibuprofen. In addition to gestational age, ethnicity was strongly associated with the response to ibuprofen and PDA closure in the present study. The awareness of ethnic/racial disparities in neonatal care of preterm infants has been growing because of considerable data gathered from recent clinical and experimental studies. For example, it is clear that there is a 2- to 3-fold racial difference in preeclampsia associated dysfunctional vasodilatation [31]. Similarly, common genetic variants in genes encoding pro- or anti-inflammatory cytokines may influence the risk for spontaneous preterm birth. It has been shown that genetic factors may also influence the susceptibility to white matter damage and subsequent cerebral palsy in very preterm infants [32]–[35]. In rodents, strains disparities have been reported to account for differences in brain vulnerability to neonatal hypoxic-ischemic insult [36]. Recently, two reports support that preterm PDA is highly familial with contribution of both genetic and environmental factors [37], [38]. Moreover, ductus remodeling, likely genetically determined, was recognized as one of the most important factors for PDA closure [39].

Ethnic disparities in neonatal and post-neonatal mortality, documented in the United States, is related to many factors both genetic and epigenetic, including health care disparities, differences in risk factors and causes of neonatal mortality, differences in drug efficacy [40]. African Americans experience a lower risk of neonatal mortality in preterm and low birth weight infants compared to white or hispanic ethnic groups [41]. In contrast, after surfactant therapy for RDS became generally available, neonatal mortality improved more for white than for black infants with very low birth weight [42]. African American infants have been shown to have a lower incidence of RDS than whites after controlling for birth and gestational age [43], [44]. This observation is consistent with the present study showing a significantly higher incidence of additional surfactant requirement in CYP2C variant infants who were tightly associated with Caucasian ethnicity. These differences have been attributed to more rapid lung maturation and consequently earlier production of pulmonary surfactant in African American foetuses compared with white foetuses [45]–[47]. Altogether, these data support that, in addition to environmental or sociodemographic factors, genetically-determined ethnic differences account for disparities in respiratory illness and response to treatment in VLBW neonates. Our study suggests that similar conclusions could be made for PDA closure and response to ibuprofen but unrelated to CYP2C genotype.

In conclusion, *CYP2C8* and *2C9* polymorphisms did not appear to be involved in PDA response to ibuprofen and cannot be used to optimize the ductal closure rate by modulating ibuprofen dosing strategy. In contrast, this study points out the role for ethnicity in the interindividual variability of response to ibuprofen and subsequent PDA closure in preterm infants. Interethnic differences in the neonatal PDA clinical course should be further explored and correlated to ibuprofen pharmacokinetics.
